# Sex work involvement among women with long-term opioid injection drug dependence who enter opioid agonist treatment

**DOI:** 10.1186/1477-7517-9-8

**Published:** 2012-01-25

**Authors:** Kirsten Marchand, Eugenia Oviedo-Joekes, Daphne Guh, David C Marsh, Suzanne Brissette, Martin T Schechter

**Affiliations:** 1 Centre for Health Evaluation & Outcome Sciences, Providence Health Care, St. Paul's Hospital 620B-1081 Burrard Street, Vancouver, BC, V6Z 1Y6, Canada; 2 School of Population and Public Health, University of British Columbia, 2206 East Mall Vancouver, BC, V6T 1Z3, Canada; 3 Centre for Addiction Research BC, University of Victoria, 2300 McKenzie Ave, Victoria, BC, V8P 5C2, Canada; 4 Northern Ontario School of Medicine, 935 Ramsey Lake Road, Sudbury, ON, P3E 2C6, Canada; 5 Centre de Recherche du Centre Hospitalier de l'Université de Montréal (CHUM), 1058 St-Denis Montréal, QC, H2X 3J4 Canada

**Keywords:** Sex work, opioid dependence, substitution treatment

## Abstract

**Background:**

Substitution with opioid-agonists (e.g., methadone) has shown to be an effective treatment for chronic long-term opioid dependency. Survival sex work, very common among injection drug users, has been associated with poor Opioid Agonist Treatment (OAT) engagement, retention and response. Therefore, this study was undertaken to determine factors associated with engaging in sex work among long-term opioid dependent women receiving OAT.

**Methods:**

Data from a randomized controlled trial, the North American Opiate Medication Initiative (NAOMI), conducted in Vancouver and Montreal (Canada) between 2005-2008, was analyzed. The NAOMI study compared the effectiveness of oral methadone to injectable diacetylmorphine or injectable hydromorphone, the last two on a double blind basis, over 12 months. A research team, independent of the clinic services, obtained outcome evaluations at baseline and follow-up (3, 6, 9, 12, 18 and 24 months).

**Results:**

A total 53.6% of women reported engaging in sex work in at least one of the research visits. At treatment initiation, women who were younger and had fewer years of education were more likely to be engaged in sex work. The multivariate logistic generalized estimating equation regression analysis determined that psychological symptoms, and high illicit heroin and cocaine use correlated with women's involvement in sex work during the study period.

**Conclusions:**

After entering OAT, women using injection drugs and engaging in sex work represent a particularly vulnerable group showing poorer psychological health and a higher use of heroin and cocaine compared to women not engaging in sex work. These factors must be taken into consideration in the planning and provision of OAT in order to improve treatment outcomes.

**Trial Registration:**

NCT00175357.

## 1. Background

Opioid dependence, frequently manifested as heroin dependence, is a chronic illness that, when untreated, can result in adverse health consequences such as blood-borne viral infections, endocarditis and drug overdoses [1,2]. Illicit opioid use is also associated with severe psychosocial problems such as homelessness, unemployment, loss of family bonds, and illegal activity [3]. Survival sex work is very common among street drug users and has been associated with increased drug related harms [4-7]. Although data indicate that women as well as men using drugs engage in sex work, women who use injection drugs are more likely to be involved in survival sex work compared to men [4,8,9].

Data suggest that women who are injection drug users (IDU) and engage in sex work present greater vulnerabilities compared to non-sex workers using injection drugs. For example, they are more likely to have unstable housing [4,10], higher rates of incarceration [4,10-12] and fewer years of education [12]. These women are also more likely to report daily injection heroin use [10], higher rates of cocaine use [4] and binge drug use [13]. These observations suggest that women who use injection drugs and engage in sex work may be more vulnerable to adverse physical and psychological consequences of injection drug use.

Chronic health conditions and infectious diseases, such as human immunodeficiency virus (HIV) infection [14,15], hepatitis C [16] and sexually transmitted infections (STI) are highly prevalent among female sex workers using injection drugs. This has been supported by studies showing that HIV risk behaviours, including sharing injection equipment [4,10,11,17] and inconsistent condom use with clients [10,17] are common risk behaviours. Moreover, a recent study found that engaging in risky injection practices (e.g., sharing injection equipment) was more likely among female sex workers with psychological distress [17], indicating an association between psychological health and disease risk. Psychological health has previously been measured among sex workers and non-sex workers accessing opioid agonist therapy (OAT). In a sample of injection drug using women accessing MMT [12], it was determined that sex workers had greater psychological symptoms including depression, anxiety, psychosis and hostility, compared to women not involved in sex work.

OAT (for example with methadone or buprenorphine) is widely considered the most effective intervention for opioid dependency [18]. OAT has been proven effective at reducing illicit drug use and illegal activities, HIV infections, as well as improving general health and psychosocial adjustment [2,18-20]. There is evidence showing that involvement in sex work may be negatively associated with OAT access and outcomes, including reduced access to care [4,21,22] and early withdrawal from a low-threshold program [23]. Moreover, a recent randomized clinical trial (RCT) comparing Heroin Assisted Treatment (HAT) to Methadone Maintenance Treatment (MMT) found female sex workers had higher illicit drug use and poorer health outcomes after 12 months of treatment relative to those not engaged in sex work [8].

The studies described above suggest that among women using injection drugs, sex work is a factor that may deter women from being engaged, retained and responding to OAT. However, the factors that are associated with engaging in sex work after entering treatment are not well understood. The present study aims to determine if health (physical, mental, social), illicit drug use and treatment retention were associated with engaging in sex work after initiating OAT in a cohort of long-term opioid injection drug users.

## 2. Methods

### Design, Setting and Participants

The North American Opiate Medication Initiative (NAOMI) was an open-label, phase III RCT comparing supervised injected diacetylmorphine (the active ingredient in heroin) and oral methadone in the treatment of long-term opioid dependence. Participants' profile, study design, methodology and results of the parent study have been published elsewhere [24-26]. Briefly, eligible participants were at least 25 years of age, with a minimum of 5 years of opioid dependence, current daily injection of opioids, at least two prior treatment attempts for opioid dependence (including at least one OAT), and no enrolment in OAT within the prior 6 months.

A total of 251 individuals were randomized to receive oral methadone (n = 111) or injectable opioids (on a double blind basis: diacetylmorphine, n = 115; hydromorphone, n = 25). Oral methadone was dispensed daily and injectable medications were administered up to three times daily under the supervision of nursing staff. Participants were also offered psychosocial services and primary care on site and all services were delivered in a patient-centred fashion [27]. Medications were provided for 12 months. Since injectable medications were not licensed for addiction treatment, an additional 3-month period was provided to taper and transition those in the injection group to other treatment modalities (primarily methadone). All participants provided written informed consent and the study was approved by the University of British Columbia/Providence Health Care and Centre de Recherche du Centre Hospitalier de l'université de Montréal research ethics boards.

### Measures

A research team, independent of the clinic services, obtained outcome evaluations at baseline and follow-up (3, 6, 9, 12, 18 and 24 months), using the European Addiction Severity Index ([EuropASI]; [28]), the Maudsley Addiction Profile ([MAP]; [29]) and health related quality of life instrument- Euroquol ([EQ5D]; [30]). For the purpose of the present study, participants were considered retained at each evaluation if they received addiction treatment on at least 20 of the 30 days in the month prior to the evaluation.

Information related to sex work was obtained from the Employment/Support Status questionnaire of the EuropASI. Participants responded dichotomously to whether or not they received money from 'Prostitution' in the prior 30 days.

### Analysis

Continuous variables were described by means, median, standard deviations and interquartile range, while frequencies and proportions summarized categorical variables. A multivariate logistic regression model estimated by generalized estimating equations (GEE) algorithm for repeated measures was used to determine factors (socio-demographic, substance use, treatment history, physical and psychological health, etc.) associated with reporting sex work at baseline. To evaluate the relationship between sex work and study variables measured during the 12 month treatment period and up to 24 months follow-up, a bivariate logistic regression analysis, adjusted by baseline sex trade involvement (i.e., a logistic regression model with baseline sex trade involvement and one additional independent variable) was used. Variables that were determined significant at p value ≤ 0.1 in bivariate analyses were included in the adjusted multivariate logistic regression model, estimated by generalized estimating equations (GEE) algorithm for repeated measures. Ethnicity, age, study site, randomization arm and treatment retention were added throughout the group variable and final model selections. Odds ratios (OR) and 95% confidence intervals (CI) were calculated. Missing observations were considered as missing in the analysis.

Only four of 154 (1.3%) men reported engaging in sex work; therefore, analyses were performed for women only. Of the 97 women entering treatment, we obtained outcome measures for 81 women at 24 months (83.5%).

## 3. Results

A total of 52 (53.6%) women receiving oral and injectable medications reported being involved in sex work in at least one of the seven research visits (Table 1). Thirteen women who were not engaged in sex work at baseline reported doing so at some point during the follow-up period, while 10 of the 52 women were consistently involved in sex work at each of the seven research visits. The multivariate analysis of factors associated with baseline sex work indicated that younger women (OR for every 5 year increase in age = .76; 95% CI = .57,1.00; p = .05) and women with less education (OR for each additional year of education = .81; 95% CI = .66,1.01; p = .055) were more likely to engage in sex work.

**Table 1:** Total number of women reporting sex trade

	Women Total N = 97			
**Sex trade**	**n with sex trade**	**% (out of total N)**	**Total N with visit**	**% (out of those with visit)**

Ever (a)	52	53.6	97	53.6

Baseline	42	43.3	97	43.3

3 months	28	28.9	89	31.5

6 months	21	21.6	86	24.4

9 months	23	23.7	88	26.1

12 months	23	23.7	92	25.0

18 months	18	18.6	87	20.7

24 months	17	17.5	81	21.0

(a) Reported ever being involved in sex trade at some point during the evaluation period

The bivariate logistic regression analysis, adjusted by baseline sex trade (Table 2), indicated that treatment retention and health related quality of life were inversely associated with sex work. Sex work was more likely among women with poorer scores in social relations, greater physical and psychological health symptoms and more days of illicit heroin, cocaine and injection drug use in the prior month. Women considered to have a high (≥ 20) or medium (9-19) number of days of injection drug use in the past 30 days were more likely to report sex work compared to women with low days (≤ 8) of injection. In addition, compared to women who injected the least amount of times per day (≤ 3), those with the most frequent daily injection (≥ 7) were more likely to report sex work in the prior 30 days. There was a suggestion that injectable treatment had a protective effect on engagement in sex work with an adjusted odds ratio of .83. However, this was not statistically significant. With only about 45 women in each arm of NAOMI, the power to detect an odds ratio of .8 is virtually non-existent.

**Table 2:** Univariate logistic regression analysis, adjusted by baseline sex trade, of variables associated with engaging in sex trade after baseline

Variable (a)	OR	p-value
	(95% CI)	
Treatment Retention: (b)	0.41	0.001
Yes vs. No	(0.24, 0.68)	

Social Relations: (c)	1.08	0.009
Every 0.2 unit increase	(1.02, 1.14)	

Days injecting drugs: (c)	6.40	< 0.001
High (≥ 20) vs. Low (≤ 8)	(3.37,12.18)	
	
	3.26	0.002
Medium (9-19) vs. Low (≤ 8)	(1.53, 6.95)	

Times injecting on a typical day: (d)	5.40	0.001
High (≥ 7) vs. Low (≤ 3)	(2.05, 14.22)	

Days with heroin use:	5.12	< 0.001
Every 5 day increase	(2.76, 9.52)	

Days with cocaine use:	5.92	< 0.001
Every 5 day increase	(2.77, 12.66)	

EQ5D: (e)	0.99	0.019
Every 0.1 unit increase	(0.98, 1.00)	

Physical health symptoms: (d)	1.05	0.02
Every 1 unit increase	(1.01, 1.09)	

Psychological symptoms: (d)	1.09	< 0.001
Every 1 unit increase	(1.06, 1.13)	

OR: Odds ratios; CI: Confidence Interval

(a) All variables refer to the prior month;

(b) Retention to treatment: at least 20 out of prior 30 days;

(c) EuropASI (European version of the Addiction Severity Index). Sub-scale scores range from 0 to 1; higher scores are indicative of more severe problems;

(d) MAP (Maudsley Addiction Profile). Scores range from 0 to 40; higher scores are indicative of more symptoms;

(e) EQ5D (Euroquol) Scores range from 0 to 1; higher scores are indicative of less severe problems; EQ5D index score with U.S. weights.

In the multivariate logistic regression GEE model (Table 3), women with more days of heroin (OR = 1.26; 95% CI = 1.05, 1.15; p = .01) and cocaine use (OR = 1.36; 95% CI = 1.16, 1.60; p < .001) and greater psychological symptoms (OR = 1.07; 95% CI = 1.03, 1.11; p < .001) in the prior month were more likely to engage in sex work compared to those with less psychological symptoms, and days of heroin and cocaine use.

**Table 3:** Multivariate GEE model of predictors of engaging in sex trade after baseline

Variable (a)	OR	p-value
	(95% CI)	
Heroin use:	1.26	0.01
Every 5 day increase	(1.05, 1.15)	

Cocaine use:	1.36	< 0.001
Every 5 day increase	(1.16, 1.60)	

Psychological symptoms: (b)	1.07	< 0.001
Every 1 unit increase	(1.03, 1.11)	

OR: Odds ratios; CI: Confidence Interval;

Model adjusted by ethnicity, interaction between age and randomization arm, treatment retention (not significant) and study site and baseline sex work (significant).

(a) All variables refer to the prior month;

(b) MAP (Maudsley Addiction Profile). Scores range from 0 to 40; higher scores are indicative of more symptoms.

## 4. Discussion

The aim of this study was to determine factors associated with engaging in sex work among long-term opioid injection drug users receiving OAT in the frame of a clinical trial. A higher proportion of women (53.6%) compared to men (1.3%), reported engaging in sex work in at least one of the seven research visits from baseline to 24 months. At treatment entry, age and education were associated with sex work, while during the study period, psychological symptoms and frequent heroin and cocaine use in the prior 30 days were associated with sex work.

At treatment initiation, women who were younger and had fewer years of education were more likely to be engaged in sex work, factors which have previously been associated with sex work among women using injection drugs [4,10,12]. Housing, ethnicity and incarcerations have also been documented in previous studies [4]. The lack of such associations in the present study reflects the homogeneity of the NAOMI sample, possibly due to study inclusion criteria.

When examining factors associated with sex work involvement during the study period, women with poorer treatment outcomes were more likely to engage in sex work. Specifically, lower treatment retention, poorer scores in social relations and health related quality of life, more days of illicit drug use, injection drug use, and more frequent daily injection in the prior 30 days. These findings indicate that sex work was more likely among a subgroup of women who did not fully benefit from OAT, a noteworthy finding considering that OAT has shown to reduce many of the harms associated with long-term heroin use [2,18]. Moreover, in the present study women who were retained successfully in OAT were less likely to be involved in sex work and therefore experienced a reduced vulnerability to harms caused by injection drug use. While this is not a causal association, it indicates that those involved in sex work were more likely to drop-out of treatment.

In the multivariate model, psychological symptoms and high illicit heroin and cocaine use in the prior 30 days were associated with sex work. Similarly, previous studies have found that sex workers accessing MMT [12] and syringe exchange programs [10] presented with higher psychological distress compared to women not engaged in sex work. In addition, a higher use of substances [13], including more frequent daily heroin and cocaine use [4,10] has also been reported among injection drug using women who also engage in sex work. The results of the present study complement prior research in the context of a prospective design that allowed us to capture predictors of sex work involvement over a 24 month study period. After engaging these participants in OAT, women who continued engaging in sex work were more likely to continue using heroin and cocaine, independent of OAT retention. Thus, many women continued engaging in survival sex work and using illicit heroin, despite that OAT improves retention and reduces illicit heroin use. The complexity of the relationship between OAT effectiveness and its impact on sex work engagement requires further study.

Education is regarded as a strong indicator of social and health-related inequalities [31], and women with fewer years of education were more likely to engage in sex work at treatment initiation. These findings indicate that women with less education experience further vulnerabilities even within a population with very low socio-economic status. Therefore, those who provide addiction treatment services must consider this special circumstance, acknowledging women's financial needs and the stigma attached to sex work, so that services and policies do not further exclude these groups.

The present study focuses on long-term opioid injection drug using women with and without involvement in sex work. It is well known that opioid-dependent individuals often show poor mental and physical health as well as poor psychosocial functioning, especially after long-term use [3,32,33]. There is also growing research evidence among women and men accessing OAT demonstrating that women enter treatment with worse physical and psychological health [8,9], as well as higher opioid and stimulant use [34,35,35]. Some evidence has also suggested that women have poorer OAT outcomes compared to men [8,9]. Therefore, women using injection drugs represent a particularly important group in the provision of effective addiction treatment.

Unexpectedly, there was no association between victimization (e.g., physical, emotional, and sexual abuse) and sex work in the present study. Previous studies have shown high rates of physical and sexual abuse in sex workers' childhood, and later victimization by partners [12,36] and clients in adulthood [37]. Moreover, in a cohort study of youth using substances, childhood sexual abuse was independently associated with sex work [38]. One possible explanation for the absence of this expected association may be related to the measure of victimization. The Addiction Severity Index (ASI) has been used to evaluate abuse in several studies [39], measuring victimization with a general question (e.g., "have you ever been physically or sexually abused?"). Therefore, [40-42] details regarding the nature of the event, which might account for the associations between victimization and sex work, are undetermined [43,44].

Limitations of the NAOMI study have been discussed elsewhere [24,25]. It should be noted that the analysis were intent-to-treat, therefore, some of the higher intensity drug use occurred in participants who were not receiving the treatments as provided in the study (oral and injectable arms). Several gender sensitive and sex work specific-related questions were not part of the study evaluation package (e.g., partner's use of illicit substances, income earned from sex work to support heroin use), that data would have provided a more detailed picture of the situation. In addition, the trial was not designed to investigate factors associated with sex work and we had a small sample size; however it provided an opportunity to obtain valuable information on this topic in the context of women receiving OAT. In order to better conceptualize the relationship between high intensity drug use and sex work, additional data regarding the reasons for sex work involvement during treatment, the proportion of earnings used from sex work to support illicit drug use, and information regarding the people who depend on an individual involved in sex work, should be captured..

The findings presented suggest that participation in NAOMI positively affected the pattern of sex work, showing a decline from enrolment to 24 months follow-up. At treatment entry, all NAOMI participants had not received any treatment for the six months prior to study enrolment (as per inclusion criteria); therefore considered un-treated despite the available options (e.g., methadone treatment). Engagement in treatment was associated with a decline in sex work over time. This particular group would have likely remained outside of addiction treatment services, and likely only initiated treatment for the opportunity to receive injectable diacetylmorphine. Certainly, approaches that improve treatment engagement (such as medically prescribed diacetylmorphine) for long-term treatment refractory heroin injectors, must be supported by current policies.

## 5. Conclusion

Findings of this study suggest that injection drug using women engaged in sex work represent a highly vulnerable group with poorer psychological health and a greater use of heroin and cocaine while receiving OAT. Future research should aim to better understand the circumstances around illicit drug use and sex work among opioid-dependent individuals' receiving OAT, as these activities impact treatment outcomes and the addiction recovery process. In addition, mixed methods studies exploring sex workers' perceptions of OAT and barriers to treatment engagement may provide valuable information for the development of future interventions and design of tailored services which should aim to simultaneously reduce the harms associated with injection drug use and sex work.

## Competing interests

The authors declare that they have no competing interests.

## Authors' contributions

MTS, SB, DM made substantial contributions to conception and design of the study; MTS, SB, DM, EOJ and DG made substantial contributions to acquisition of data, and analysis and interpretation of data; KM made substantial contributions to analysis and interpretation of data. The first (KM), second (EOJ) and last author (MTS) wrote the first draft of the paper, the senior statistician (DG) performed the data analyses. All authors critically revised the manuscript for important intellectual content. The final decision about publishing the paper was made by all the authors.
